# Nephrocalcinosis in very low birth weight infants: incidence, associated factors, and natural course

**DOI:** 10.1007/s00467-021-05417-w

**Published:** 2022-03-28

**Authors:** Jeanne Fayard, Pierre Pradat, Sylvie Lorthois, Justine Bacchetta, Jean-Charles Picaud

**Affiliations:** 1 Department of Neonatology, Hopital de la croix rousse, Hospices Civils de Lyon, 69004 Lyon, France; 2 Department of Neonatology, Hôpital de La Conception, Assistance Publique Des Hôpitaux de Marseille, 13005 Marseille, France; 3 Centre for Clinical Research, Hopital de la croix rousse, Hospices Civils de Lyon, 69004 Lyon, France; 4 Department of Pediatric Radiology, Hôpital Femme Mère Enfant de Lyon, 69677 Hospices civils de Lyon, Bron, France; 5 Reference Center for Rare Diseases of Calcium and Phosphate Metabolism, Pediatric Nephrology Rheumatology and Dermatology Unit, Hôpital Femme Mère Enfant de Lyon, 69677 Hospices civils de Lyon, Bron, France; 6 INSERM 1033 Research Unit and Lyon, Est Medical School, Lyon 1 University, 69008 Lyon, France; 7 CarMen Laboratory, INSERM, INRA, Claude Bernard University Lyon 1, 69310 Pierre Bénite, France

**Keywords:** Prematurity, Diuretics, Hypercalciuria, Growth, Kidney function, Vitamin D

## Abstract

**Background:**

Preterm kidney is exposed to various exogenous factors that may impact its function such as nephrotoxic drugs or nephrocalcinosis. We investigated prevalence and risk factors of nephrocalcinosis (NC) in recently born very low birth weight (VLBW) infants submitted to improved biological monitoring.

**Methods:**

Retrospective, case–control study in very preterm infants (< 32 + 6 weeks, ≤ 1500 g) admitted to a tertiary care unit during a 6-year period. Each case (ultrasound-diagnosed NC) was matched with two controls (no NC). Data were collected at days 15 and 30 of life and 35 weeks corrected age, with follow-up at 18 months and 3 years.

**Results:**

Of 525 eligible infants, overall prevalence of NC was 17.1% at 35 weeks corrected age. Prevalence was halved between 2012 (26.1%) and 2017 (11.8%). We included 265 infants, more than half being born before 28 weeks. Cases presented with more severe morbidity than controls, but reached statistical significance only in infants born < 28 weeks (88.2% vs. 68.3%, *P* = 0.01). Protein, energy, calcium, phosphorus, and vitamin D intakes were similar in the two groups and did not change significantly over the study period. Weight gain was similar in the two groups. Exposure to furosemide (OR [IC95%]: 1.26 [1.02; 1.57]) and postnatal growth (1.65 [1.04; 2.67]) were independent risk factors of NC. NC resolved 12–18 months after diagnosis in 61% of infants.

**Conclusion:**

Prevalence of NC is significant but can be reduced. Furosemide should be cautiously prescribed in VLBW infants, and nutritional support must be well monitored to support postnatal growth and limit risk of nephrocalcinosis.

**Trial registration:**

ClinicalTrials.gov: NCT 04,860,583.

**Graphical abstract:**

A higher resolution version of the Graphical abstract is available as Supplementary information

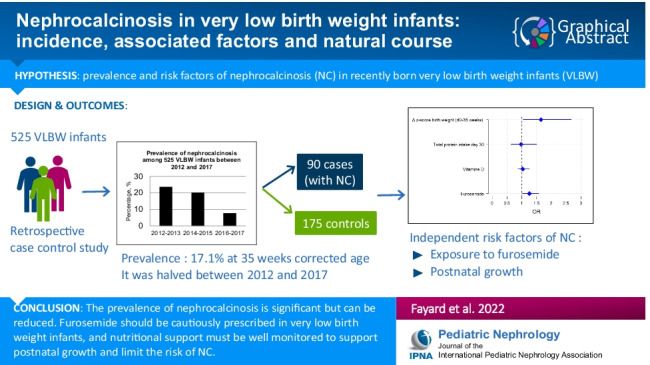

**Supplementary Information:**

The online version contains supplementary material available at 10.1007/s00467-021-05417-w.

## Introduction

Besides prematurity itself [1, 2], the preterm kidney is exposed to various exogenous factors after birth that may impact its function, such as nephrocalcinosis (NC) among others [2–10]. In neonates, NC development is associated with hypercalciuria or more rarely with enteric hyperoxaluria [11–15]. It was first described in low birth weight (BW) infants more than 25 years ago [16]. There are huge discrepancies in reported NC rates, ranging from 7 to 64% [11, 15, 17–25]. This is likely related to the type of population studied, variations in practices in neonatal units, and ultrasound inter-operator variability.

NC has been associated with long-term renal complications such as increased blood pressure, altered tubular or glomerular functions, and reduction in kidney growth and volume [2, 4, 5, 24, 26, 27].

The etiology of NC in preterm infants is still imperfectly known, as it is likely multifactorial. The main known risk factors are as follows: low gestational age (GA) (< 32 weeks), low birth weight (< 1500 g), prolonged oxygen supplementation or assisted ventilation, prolonged parenteral nutrition, postnatal drugs (furosemide, glucocorticoids), inadequate mineral intake [5–7, 11, 21, 25, 28–30]. Unfortunately, previous studies had significant methodological limits: small number of patients, lack of control groups, small proportion of extremely preterm infants, and different care to what is currently done (antenatal steroids, parenteral and enteral nutrition, human milk fortification).

As there was no consensus on kidney follow-up of preterm infants, we set up in 2009, a renal monitoring protocol for all preterm infants born under 32 weeks and/or under 1500 g, including kidney ultrasound at 35 weeks corrected GA.

We aimed to investigate the prevalence and risk factors of NC in a population of recently born very low birth weight (VLBW) infants including a significant proportion of extreme preterm infants.

## Population and methods

### Type of study

We performed a retrospective, case–control study.

### Patients

We included preterm infants born between January 1, 2012, and December 31, 2017, with a GA ≤ 32 + 6 weeks and a BW ≤ 1500 g. We chose a 6-year period so that the study was sufficiently long to reliably assess NC prevalence. Considering that 220 to 250 VLBW infants are admitted to our unit each year, we were expecting a population of 1300 to 1500 infants. We included infants admitted before day 3 of life in the tertiary care unit at Croix–Rousse hospital, who stayed in the unit until discharge home. Neonates with congenital malformations were excluded from the study. Cases had a diagnosis of NC at kidney ultrasound performed at 35 weeks corrected GA. NC was considered when increased medullary echogenicity was found. Each case was matched with two controls (no NC at 35 weeks) according to the following criteria: year of birth, GA, antenatal steroids, and intra-uterine growth restriction (IUGR).

### Feeding regimen

In our unit, parenteral nutrition was started from the first hours of life and was stopped when enteral intake was well-tolerated and reached 100 to 120 ml·kg^−1^·day^−1^. Enteral feeding was started as soon as possible after birth using unfortified pasteurized donor human milk. A multicomponent fortifier was added to human milk (mother’s own milk or donor human milk) when 70 ml·kg^−1^·day^−1^ enteral intake was well-tolerated. When full enteral feeding (160 ml·kg^−1^·day^−1^) was reached, individualized adjustable fortification of human milk was performed, based on the monitoring of weight gain and serum urea. Infants received human milk up to 1800 g. Then, infants were fed with either fortified human milk or a preterm formula until discharge. According to French recommendations, infants received a daily oral multivitamin supplement providing 1000 IU of vitamin D per day, starting when enteral feeding was initiated.

### Nutritional and renal monitoring

In 2009, we implemented a renal monitoring protocol for VLBW infants, including kidney ultrasound at 35 weeks corrected GA. Then we progressively improved biological monitoring including serum and spot urine sodium (Na), calcium (Ca), phosphorus (P), urea, and creatinine (Cr) every 2 weeks to adapt protein and mineral intake. We aimed for weight gain of 20 g·kg^−1^·day^−1^. When this was not reached, we considered serum urea. If it was low (< 3 mmol/L), protein intake was increased. When serum urea was above 3 mmol/L, we increased energy intake. Calcium and phosphorus intakes were adapted aiming for a urinary Ca/Cr ratio < 2.2. We progressively added the assessment of serum 25(OH)D at 1 month of life and then once a month, aiming for a value between 50 and 120 nmol/L. This monitoring protocol allowed us to more precisely adjust nutritional intakes to obtain a better postnatal growth and avoid hypercalciuria which favors NC.

### Data collected

We collected the following: GA, anthropometric measurements at birth (weight, crown-heel length, head circumference), gender, antenatal steroids, length of stay at hospital, anthropometric measurements at discharge, IUGR defined as birth weight below the third percentile of Fenton reference values, and kidney ultrasound results at 35 weeks corrected GA [31]. Kidney ultrasound was performed by a single pediatric radiologist (SL) using a convex 8C-RS (4–11 MHz) and a linear probe 12L-RS (6–13 MHz) on a VIVID-E (General Electric, Boulogne-Billancourt, France). The following data were collected at days 15 and 30 of life and 35 weeks: duration of invasive and non-invasive ventilation, drugs used (caffeine, diuretics, steroids, vitamin D, aminoglycosides), and duration of exposure to these drugs between birth and 35 weeks. For diuretics and postnatal steroids, we calculated the exposition rate, the total duration of exposure between birth and 35 weeks. The postnatal steroid (betamethasone or hydrocortisone hemisuccinate) dose was expressed in milligrams and milligrams per kilogram equivalent prednisone. We also collected data about clinical outcomes during the hospital stay: neurological disease (intraventricular hemorrhage), retinopathy of prematurity, bronchopulmonary dysplasia (BPD), necrotizing enterocolitis, and late-onset sepsis. A composite index of severe morbidity was considered present when at least one criterion existed among treated patent ductus arteriosus, late-onset sepsis, necrotizing enterocolitis stage ≥ 2, moderate or severe BPD, severe retinopathy of prematurity (stage 2 plus, stage 3, laser photocoagulation or anti-VEGF treatment), intraventricular hemorrhage ≥ grade 3, or periventricular leucomalacia.

Type and quantity of milk (human milk or formula) were recorded as well as the duration of parenteral nutrition. At each time point (day 15, day 30, 35 weeks), the mean intakes per kilogram per day of Ca, P, vitamin D, protein, energy, protein/energy ratio, and Ca/P ratio were calculated.

Anthropometric data were collected at each time point and at discharge. Weight-for-age, length-for-age, and head circumference-for-age *z*-scores were calculated using Fenton reference values [31]. To precisely evaluate postnatal growth, the differences in *z*-scores between birth and 35 weeks were also evaluated. Measurements of Na, P, Ca, Cr, and urea in blood samples and spot urine samples were taken at each time point. The first blood sample to assess serum electrolytes, urea, and creatinine was collected at 24 h of life, according to our unit protocol. We analyzed the decrease in serum Cr between birth and day 15 of life, as it could reflect the speed of establishment of the infants’ own kidney function. Urinary Ca excretion was expressed both in absolute value and using the ratio Ca/Cr.

As these high-risk infants participated in a follow-up after discharge, we were able to collect anthropometric data at 18 months and blood pressure values at 3 years.

### Statistical analysis

#### ***Sample size***

The inclusion of at least 80 infants with NC and twice as many controls would allow us to detect a 20% difference in exposure rate (deemed as clinically relevant) between cases and controls with a power of 83% (p1 = 60%, p2 = 40%, alpha risk 5%).

Matching of each NC case to two controls based on year of birth, GA, IUGR, and antenatal steroids was done using the nearest neighbor method, which is a multivariate logistic model based on the four variables. This method is based on the propensity score principle. For each patient, the probability of being a case is calculated according to the four variables above and the choice of controls is made according to this probability (the case and its two matched controls will have close probabilities). This matching was done using the “MatchIt” package of the R software.

Qualitative variables are described in numbers and percentages, and the quantitative variables in median and interquartile ranges (IQR25, IQR75). For the qualitative variables, comparisons between groups were made using the Chi^2^ test or the Fisher exact test for small numbers (at least one notional number < 5). For quantitative variables, inter-group comparisons were made using Student’s *t*-test for normally distributed variables or the non-parametric Mann–Whitney test for non-normally distributed variables. For each quantitative variable, normality was tested using the Shapiro–Wilk test.

Potential associations between quantitative variables were tested using the Pearson correlation coefficient. Multivariate logistic regression analysis was performed. Variable selection was made using the “glmulti” function (“glmulti” package from the R software), which, from a list of explanatory variables, looks for the best model using the Akaike information criterion (AIC). The results of the multivariate analysis are expressed in odds ratios (OR) with a 95% confidence interval (95% CI). A value of *p* < 0.05 was considered statistically significant. All analyses were performed using R software (R Foundation for Statistical Computing, Vienna, Austria).

### Ethics approval

The study was approved by the Ethical Committee of Hospices Civils de Lyon (HCL) and declared to the National Committee on Data Collection (Commission Nationale Informatique et Liberté, CNIL) (HCL CNIL registered under number 19–189). The study has been registered on ClinicalTrials.gov: NCT 04,860,583.

## Results

**Fig. 1:**
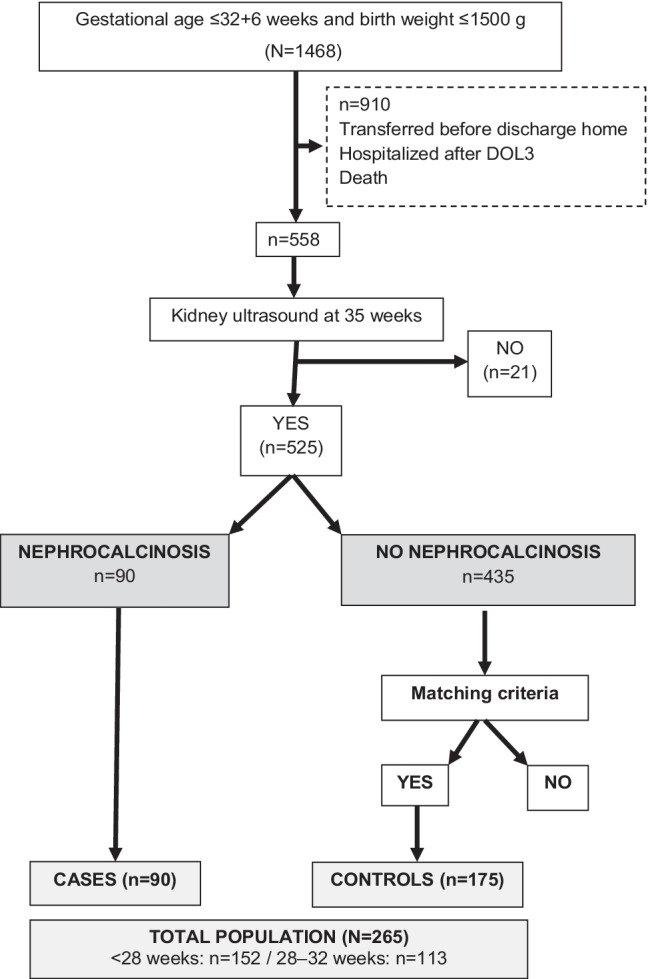
Flowchart. Analysis of nephrocalcinosis in 525 very premature infants between 2012 and 2017

Over the 6-year study period, 525 infants were eligible (Fig. 1). About one infant out of six (*n* = 90/525, 17.1%) presented a NC at 35 weeks. This prevalence was higher in males than in females (54/251 = 21.5% vs. 36/274 = 13.1%, respectively; *p* = 0.011). Most cases (56.7%) were diagnosed in infants born before 28 weeks. Infants with NC (cases) were matched with 175 controls. Therefore, 265 infants were included, more than half of whom were born before 28 weeks (*n* = 152/265, 57%).

**Fig. 2:**
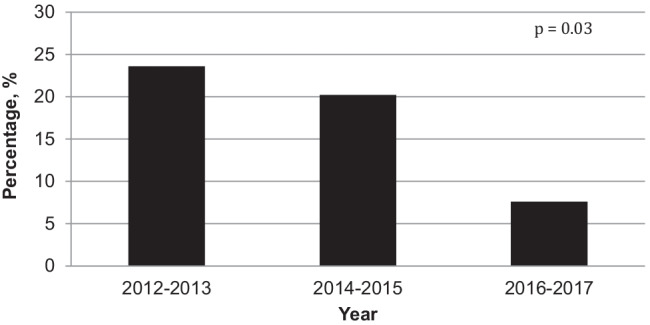
Prevalence of nephrocalcinosis among 525 very premature infants between 2012 and 2017 (percentage, %)

The prevalence of NC was twice as high in infants born before 28 weeks (26.1%) than in infants born at 28–32 weeks (11.8%, *p* < 0.001). The prevalence of NC decreased by more than half between 2012 (26%) and 2017 (11%, *p* = 0.03) (Fig. 2).

Characteristics at birth were similar in cases and controls (Table 1). Birth weight ranged from 440 to 1490 g and GA from 23 + 5 weeks to 32 + 4 weeks. The sex ratio was similar. About one infant out of four had a *z*-score for birth weight below –1 SD (cases: 23.3%, controls: 25.1%, *p* = 0.75), and infants with a *z*-score below –2 SD were scarce (3.3% vs. 2.3%, *p* = 0.69). The median duration of respiratory support was similar in both groups: about 1 week (7.3 vs. 5.6 days, *p* = 0.18) for assisted ventilation and about 1.5 months (45.6 vs. 49.7 days, *p* = 0.96) for non-invasive ventilation. Serum Cr at day of birth was similar in both groups (74 vs. 76 µmol/L, *p* = 0.21). Between birth and 35 weeks, exposure to furosemide tended to be more frequent in cases than in controls (70% vs. 58.3%, *p* = 0.06). There was no significant difference in postnatal steroid administration, and the median total dose was similar in cases and controls (10.9 vs. 11.4 mg prednisone equivalent, *p* = 0.68). The prevalence of complications related to prematurity was similar in both groups (morbidity index: 70% vs. 67.4% of infants, *p* = 0.67), except for NEC grade ≥ 2 (0% vs. 7.4%, *p* = 0.01). Cases presented with more severe morbidity than controls, but this did not reach statistical significance (61.1% vs. 49.7%, *p* = 0.08) except in infants born before 28 weeks (88.2% vs. 68.3%, *p* = 0.01) (Supplementary Table 2). In infants born between 28 and 32 weeks, cases more frequently received postnatal steroids than controls (*p* = 0.04) (Supplementary Table 2).

**Table 1:** Characteristics of 265 infants included between 2012 and 2017

	Controls	Cases	*p*
*N*	175	90	
Antenatal steroids, *n* (%)	164 (93.7)	84 (93.3)	0.90
GA (weeks)	27.7 (26.4–29.1)	27.5 (26–29.1)	0.88
BW (g)	900 (760–1140)	980 (760–1128)	0.74
Male gender, *n* (%)	87 (49.7)	54 (60)	0.11
Length (cm)	35 (33–37.5)	35 (33–37)	0.57
HC (cm)	24.7 (23.2–26.5)	25 (23.3–26.5)	0.5
*Z*-score BW	–0.3 (–1; 0.1)	–0.3 (–1; 0.1)	0.98
* Z*-score BW < -1DS, *n* (%)	44 (25.1)	21 (23.3)	0.75
Z-score BW < -2DS, *n* (%)	4 (2.3)	3 (3.3)	0.69
*Z*-score length	–0.2 (–0.9; 0.4)	–0.4 (–0.8; 0.2)	0.34
*Z*-score HC	–0.2 (–0.8; 0.3)	–0.1 (–0.7; 0.5)	0.25
Vitamine D intake (UI/d)	781 (647–866)	780 (680–852)	0.82
Caffeine (d)	41 (31–52.8)	43.5 (30–55)	0.94
Aminoglycosides (d)	2 (2–4)	2 (2–4)	0.94
Furosemide, *n* (%)	102 (58.3)	63 (70)	0.06
Furosemide (d)	3 (1–7)	3 (1.5–8.5)	0.54
Postnatal steroids, *n* (%)	33 (18.9)	24 (26.7)	0.14
Postnatal steroids duration (d)	8 (4–19)	6 (3–10.8)	0.32
Parenteral nutrition (days)	13 (9–19)	12 (8–16.8)	0.41
Human milk (mL/day)	123 (84–225)	157 (91–237)	0.15
Formula (mL/day)	112 (12–190)	108 (16–188)	0.88
PDA treated, *n* (%)	65 (37.1)	41 (45.7)	0.19
Cerebral lesions, *n* (%)	11 (6.3)	10 (11.1)	0.17
BPD, *n* (%)	73 (41.7)	48 (53.3)	0.16
NEC ≥ 2, *n* (%)	13 (7.4)	0 (0)	0.01
Late-onset sepsis, *n* (%)	77 (44)	41 (45.6)	0.81
ROP, *n* (%)	24 (13.7)	15 (16.6)	0.58

Values are median (IQR25%–IQR75%) or number (percentage)

*GA*, gestational age; *BW*, birth weight; *HC*, head circumference; *PDA treated*, patent ductus arteriosus treated medically or surgically; *Cerebral lesions*, intraventricular hemorrhage grades 3–4 or periventricular leukomalacia; *BPD*, bronchopulmonary dysplasia; *NEC*, necrotizing enterocolitis; *ROP*, retinopathy of prematurity; *Formula*, cow’s milk-based formula

**Table 2:** Characteristics of 152 infants born before 28 weeks and 113 infants born between 28 and 32 + 6 weeks

	GA < 28 weeks			GA = 28–32 weeks		
	Controls	Cases	*p*	Controls	Cases	*p*
	*N* = 101	*N* = 51		*N* = 74	*N* = 39	
Therapeutics						
Aminoglycosides (d)	4 (2–5)	4 (2–4)	0.57	2 (0–2)	2 (0–2)	0.46
Caffeine (d)	51 (45–62)	54 (45–58)	0.74	31 (20–34)	29 (21–34)	0.78
Furosemide, *n* (%)	83 (82.2)	47 (92.2)	0.09	19 (25.7)	16 (41)	0.09
PNS, *n* (%)	33 (32.7)	21 (41.2)	0.3	0 (0)	3 (7.7)	0.04
Morbidity						
PDA treated, *n* (%)	60 (59.4)	36 (70.6)	0.18	5 (6.8)	5 (12.8)	0.31
Brain lesions, *n* (%)	8 (7.9)	7 (13.7)	0.26	3 (4.1)	3 (7.7)	0.41
BPD, *n* (%)	64 (63.4)	41 (80.4)	0.1	9 (12.2)	7 (17.9)	0.7
NEC ≥ 2, *n* (%)	7 (6.9)	0 (0)	0.1	6 (8.1)	0 (0)	0.09
LOS, *n* (%)	60 (59.4)	32 (62.7)	0.73	17 (23)	9 (23.1)	1
MI, *n* (%)	87 (86.1)	49 (96.1)	0.06	31 (41.9)	14 (35.9)	0.54
Severe MI, *n* (%)	69 (68.3)	45 (88.2)	0.01	18 (24.3)	10 (25.6)	0.88
Urinary test						
Ratio Ca/creatinine	1.3 (0.9–2.3)	1.5 (1–2.2)	0.70	1.2 (0.9–2.1)	1.5 (1–2)	0.71
Ca > 3.8, *n* (%)	2 (25)	6 (75)	0.05	2 (66.7)	1 (33.3)	0.60

Values are median (IQR25%–IQR75%) or number (percentage)

*GA*, gestational age; *PNS*, postnatal steroids; *PDA treated*, patent ductus arteriosus treated medically or chirurgically; *Cerebral lesions*, intraventricular hemorrhage grades 3–4 or periventricular leukomalacia; *BPD*, bronchopulmonary dysplasia; *NEC*, necrotizing enterocolitis grade ≥ 2; *LOS*, late-onset sepsis; *MI*, morbidity index

The median duration of parenteral nutrition was around 15 days and was similar in the two groups. Protein, energy, Ca, P, and vitamin D intakes at day 15, day 30, and 35 weeks corrected GA were similar in the two groups. This intake did not change significantly between 2012 and 2017 (Supplementary Fig. 3). Only protein intake at day 30 showed variation depending on the year, with a slightly higher intake in 2015 and 2016 (Table 2).

**Fig. 3:**
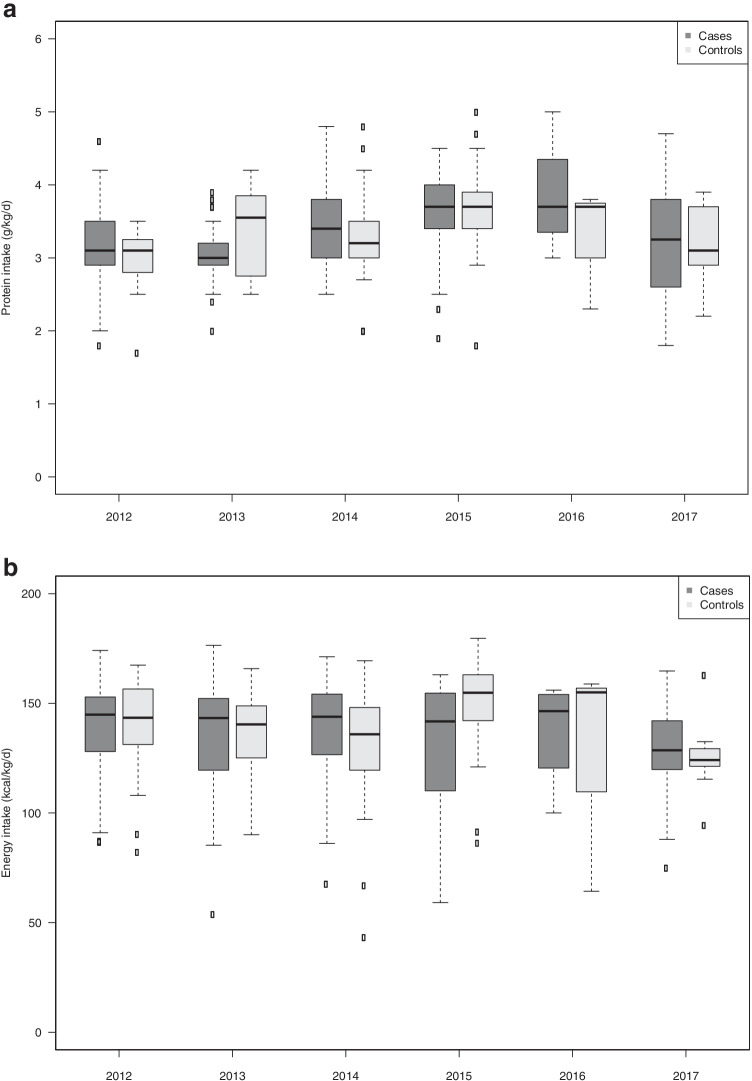

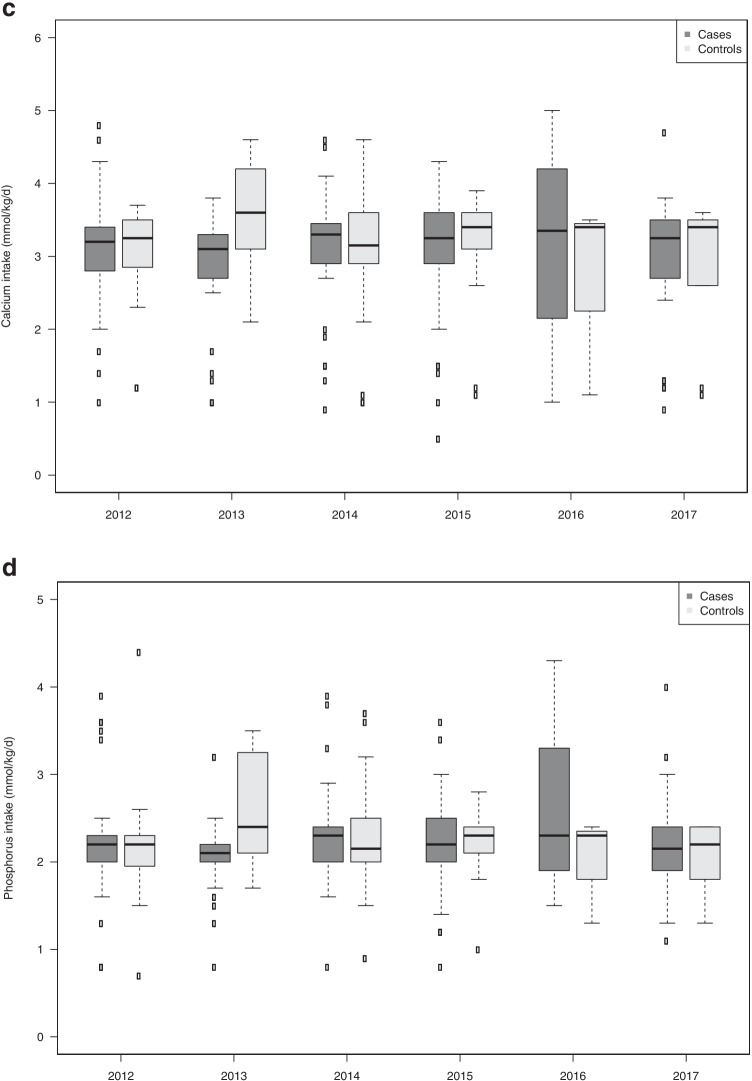
Protein (**a**), energy (**b**), calcium (**c**), and phosphorus (**d**) intake at the end of the first month of life in cases and in controls, during the study period 2012–2017

Weight gain between birth and 35 weeks corrected GA was about 15 g/kg/day and was similar in the two groups (*p* = 0.24). Similarly, the differences in *z*-score for body weight between birth and 35 weeks as well as growth up to 18 months corrected age were similar in the two groups (Table 3). Difference in *z*-score for crown-heel length between birth and 35 weeks was lower in cases than in controls, without statistically significant difference (–1 DS vs. –1.2 DS, *p* = 0.05).

**Table 3:** Postnatal growth at 36 weeks and 18 months corrected age in 265 very preterm infants hospitalized between 2011 and 2017

	Controls	Cases	*p*
***N***	175	90	
At 36 weeks			
GA (weeks)	36 (35.3–37)	36 (35.3–37)	0.73
Postnatal age (d)	59.5 (46.3–72.8)	63 (46–75)	0.67
Weight (g)	2325 (2010–2698)	2360 (2068–2723)	0.24
ΔW d30–35 weeks (g/kg/d)	16.3 (14.3–18)	16.4 (15.2–19)	0.09
ΔW birth–35 weeks (g/kg/d)	14.3 (13.2–15.4)	14.6 (13.4–15.7)	0.24
Body weight *Z*-score	–0.8 (–1.5; –0.3)	–0.6 (–1.1; –0.1)	0.13
Weight SDS < –1DS, *n* (%)	71 (40.6)	28 (31.1)	0.16
Weight Δ SDS (d0–35 weeks)	–0.4 (–0.8; –0.1)	–0.3 (–0.7; 0)	0.09
Weight loss d0–35 weeks > 1DS, *n* (%)	24 (13.7)	10 (11.1)	0.58
At 18 months			
Postnatal age (d)	644 (623–666)	649 (626–664)	0.62
Weight (kg)	10.0 (8.9–10.6)	10.2 (9–10.8)	0.11
Length (cm)	80 (78–82)	81 (79–82)	0.25
HC (cm)	47 (46–48)	48 (46–49)	0.09

Values are median (IQR25%–IQR75%) or number (percentage)

*GA*, gestational age; *ΔW*, weight gain; *HC*, head circumference; *SDS*, *z*-score for gestational age

The decrease in serum Cr value between birth and day 15 was similar between the two groups. Two hundred and forty-one spot urinary samples were analyzed during the study period. The urinary Ca/Cr ratio at day 30 was higher in cases than controls, but reached statistical significance only in infants born between 28 and 32 weeks (*p* = 0.01). During hospitalization, the absolute value of calciuria exceeded the crystallization threshold of 3.8 mmol/L more frequently in cases than controls in infants born before 28 weeks (75% vs. 25%, *p* = 0.05) (Table 2).

Exposure to furosemide (OR [IC95%]: 1.26 [1.02; 1.57], *p* = 0.035) and difference in *z*-score for body weight between birth and discharge (OR [IC95%]: 1.65 [1.04; 2.67], *p* = 0.038) were independent risk factors of NC at 35 weeks corrected GA. No correlation was found between vitamin D or protein intake and NC (Table 4).

**Table 4:** Multivariate analysis: variables associated with the occurrence of nephrocalcinosis in 265 preterm infants born before 32 + 6 weeks of gestation

	OR	95% CI	*p*
Furosemide	1.26	[1.02–1.57]	0.035
Vitamin D (per 100 UI)	1.03	[0.86–1.25]	0.748
Total protein intake day 30 (per 1 g/kg)	0.98	[0.63–1.51]	0.922
Δ *z*-score body weight (d0–35 weeks)	1.65	[1.04–2.67]	0.038

OR, odds ratio; *CI*, 95% confidence interval

After discharge, NC resolved 12–18 months after the diagnosis in about two infants out of three (61%). At 3 years of age, blood pressure was normal and similar in the two groups.

## Discussion

In a population of high-risk VLBW infants including half of extremely premature infants, NC was present in about one infant out of six. After progressive implementation of more stringent biological monitoring, NC prevalence progressively decreased from 26 to 11%. We identified exposure to furosemide and good postnatal growth as independent risk factors of NC in this high-risk population.

The prevalence of NC in our population was quite low, as NC has previously been reported in 7 to 64% of preterm infants [11, 15, 17–25]. This could be related to differences in the population characteristics and to improvement in practices during the past decades. For example, antenatal steroids were administered to almost all of our patients’ mothers, compared to only one-third of the mothers in previous studies [24]. Also, duration of invasive ventilation was nearly three times shorter than that reported by Short et al. (15 days vs. 41 days) [18]. Nevertheless, the level of morbidity was still significant in our population. The progressive decrease in NC prevalence during the study period is unlikely to be explained by changes in care during the study period. Protein, energy, mineral and vitamin D intake, and exposure to furosemide and steroids did not change significantly between 2012 and 2017. Rather, the decrease could be related to the progressive improvement in biological monitoring resulting in an improved day-care adaptation of mineral and vitamin D intake between 2012 and 2017.

Our secondary objective was to determine the risk factors for NC, to better define the target population that could benefit from prevention strategy. As it is already well-known that low GA and low birth weight are associated with an increased risk of NC, we included these two parameters in the matching criteria to more specifically analyze the other parameters [18, 25, 28]. However, we still found an influence of GA at birth, as we observed twice as much NC in infants born before 28 weeks (26.1%) than in those born afterward (11.8%).

Diuretics are commonly used, either during the first weeks of life or to decrease interstitial lung water and improve respiratory function in preterm infants with BPD [32]. In our population, about half of controls were exposed to furosemide, in agreement with previous publications [33]. We observed that exposure to diuretics remains a significant risk factor of NC, although duration of furosemide was rather short (3 days) in our population [15, 25, 34]. Gimpel et al. reported that a cumulative dose of furosemide higher than 10 mg/kg increased the risk of NC 38-fold [34]. As the usual dose is 1 mg/kg/day in our unit, this represents about 10 days of treatment. Only furosemide treatment, and no other diuretics, was independently associated with NC in our study. As furosemide is used in the majority of preterm infants with BPD [32], this could explain why BPD was previously identified as a risk factor for NC [15, 21, 28, 30].

High-quality nutritional care is essential for health and long-term development, as the absence of a deficit in postnatal growth has been associated with better cognitive development and better kidney function [10, 35]. A new risk factor, i.e., postnatal growth, was identified in our population. Weight gain between birth and 35 weeks was about 14 g/kg/day, which roughly corresponds to the commonly accepted objective in VLBW infants [31]. As *z*-scores are recommended to carefully analyze postnatal growth [36], we calculated the difference in *z*-score for body weight between birth and 35 weeks, which appeared as an independent risk factor of NC. Positive delta *z*-score for body weight is unlikely related to water overload as the same postnatal growth kinetic was observed for crown-heel length, suggesting that growth was related to the accretion of new tissues (fat, muscle, etc.) rather than to an excess of water. Previous studies did not report such results as nutritional intake, and growth was not evaluated in such a large population of very preterm infants. This result suggests that currently recommended nutritional management supports adequate postnatal growth, but could increase the risk of NC, requiring enhanced biological monitoring. It opens perspectives for future studies to find out if this association is coincidental or if nutritional intake needs to be more carefully managed to support postnatal growth without being deleterious for the kidneys. In our population, intake of nutrients, including vitamin D, corresponded to current European recommendations [37] and was similar between the two groups during the study period. However, the available biological data in our population study showed that more infants had hypercalciuria in cases than in controls, which favors NC [13–15, 38, 39]. In adults, excess protein (and sodium) intake leads to hypercalciuria [40]. This has not been reported in preterm infants, probably because high protein intake is associated with appropriate energy intake and, therefore, used mostly to support efficient protein retention and high growth rates [37]. Similarly, most preterm infants fed according to recommendations receive enteral Ca/P ratio that supports bone mineralization and supposedly avoids hypercalciuria [20]. Excessive Ca intake has been associated with hypercalciuria in preterm infants [6, 11, 41]. Narendra et al. did not find any association between hypercalciuria and NC in preterm infants, but urinary dosage was performed only once and, lately, at term corrected age [21]. In our study, hypercalciuria could have been related to an imbalance between Ca and P intake, or to hypervitaminosis D that we were unable to detect because of limited serum vitamin D assessment. However, it is more likely due to frequent exposure to furosemide. In our population, the real vitamin D intake was close to 800 IU/day in both groups, in agreement with current European recommendations [37]. One of the limitations of our study is that, between 2012 and 2017, we measured serum vitamin D mainly during the last years of the study period and, therefore, we cannot conclude that hypercalciuria observed in cases is due only to furosemide treatment. Nevertheless, in 2017, Vierge et al. reported that some preterm infants can present an exaggerated response to vitamin D supplementation that is responsible for hypercalcemia, hypercalciuria, and NC [42]. This underlines the importance of serum vitamin D assessment in the biological follow-up of premature infants. In summary, we identified furosemide as a remaining risk factor of NC and cannot make any conclusions about the impact of nutritional factors in some infants.

Contrary to previous studies, we did not confirm that parenteral nutrition, caffeine, antibiotics, and postnatal steroids were significant risk factors [15, 21, 43]. This is despite the fact that we carefully investigated these factors by analyzing not only the presence or absence of exposure, as in former studies, but also the duration of exposure.

We observed an association between severe morbidity and the occurrence of NC in the subgroup of the most immature premature children (< 28 weeks). These infants are sicker, requiring intensive care, including prolonged invasive ventilation, postnatal steroids, and diuretics for the management of BPD [18, 26]. This corroborates Chang et al.’s findings that low BW and postnatal steroid therapy were NC-independent risk factors in their multivariate analysis [25].

There are still discussions about the long-term consequences of NC on kidney function. In our population, biological indicators of kidney glomerular function were similar in children with and without NC during the hospital stay. We did not study the evolution of kidney function at follow-up. Two studies did not show any alteration of kidney glomerular function at 3 months of age and 7.5 years [5, 24]. Comparing formerly preterm infants to a control group of healthy term newborns at 7 years of age, Rakow et al. showed a trend toward a decrease in GFR (remaining normal), without significant differences [2]. However, in the very long term, this slight difference may become clinically relevant in adulthood. Thus, NC might be an aggravating factor in the specific context of prematurity characterized by an increased risk of long-term chronic kidney disease [3].

Nephrocalcinosis has previously been associated with arterial hypertension in former preterm infants [27]. Schell-Feith et al. reported that hypertension was more common at 1 and 2 years of age as compared to a population of healthy infants born at term [27]. Because they did not make a comparison with preterm infants without NC, it was not possible to evaluate the respective implication of NC and prematurity itself in later hypertension [27]. In our population, we did not find any difference in blood pressure between premature infants with and without NC at 3 years of age. This is in agreement with previous studies [2, 5].

According to available literature, a spontaneous resolution of NC occurs in most cases, i.e., 50% of infants at 1 year of age and 75% at school age [2]. In our population, NC resolved in around two-thirds (61%) of infants at 12–18 months of age. This is in agreement with Scheill-Feith et al., who reported NC in 34% and 15% of former preterm infants, respectively, at 15 and 30 months of age [27]. Although the spontaneous resolution of NC occurs in most cases, data are lacking about long-term kidney function in preterm infants who presented NC during a few months in this critical period of development.

A limitation of our study is the monocenter, retrospective design, which may limit its external validity. The study population includes a significant proportion of high-risk, very immature infants, which differs from previous studies and is representative of current populations of preterm infants in NICUs. Our practices are adapted to this population and consistent with recommendations, allowing us to reach the objective of postnatal growth close to the fetal growth rate. Previous retrospective studies about NC in preterm infants often included a moderate number of subjects and were purely observational. In our study, we matched one case with two controls, allowing for a comparison between infants with NC and others, and relevant statistical analysis to investigate risk factors of NC. Furthermore, we were able to include a significant number of patients, as the study period was sufficiently long and data collection was uniformly and prospectively performed through our patient software. However, the retrospective design did not allow for a complete analysis of all clinical and biological risk factors for NC. For example, we were not able to provide some data of interest such as detailed nutrient intake, urinary data, or complete growth and blood pressure follow-up. There were no—and there are not yet—clear recommendations for renal follow-up of preterm infants. Accurate kidney monitoring during hospitalization and the first years of life is necessary for preterm infants and should include specific measures for screening and follow-up of children with NC. Positive delta *z*-score for body weight is unlikely related to water overload as the same postnatal growth kinetic was observed for head circumference and crown-heel length; it is more likely that postnatal growth in our study population was related to the accretion of new tissues (fat, muscle, etc.) rather than to an excess of water. Delta *z*-score was greater in cases than controls without significant difference in univariate analysis (0.2 vs. 0, *p* = 0.3 for head circumference and –1.3 vs. –1.4, *p* = 0.64 for length).

Another limitation of our study is that the association between NC and enhanced postnatal growth is a post hoc result and is not the confirmation of a research hypothesis. This association should thus be confirmed in further trials.

In conclusion, the prevalence of NC in our study was 17.1% and was halved during the study period, likely due to the progressive improvement of biological monitoring. We confirmed the well-known risk factor of furosemide exposure and reported a new one: postnatal growth. The long-term consequences of NC are still unknown, but we observed that NC persists for several months in one-third of VLBW infants. Therefore, it seems important to (1) use furosemide with caution in this population, particularly if it is used in high doses or for an extended duration; (2) perform regular biological monitoring to adapt treatments and nutrient intake; and (3) screen for NC at the end of the hospital stay. In future prospective studies, in large populations, informative biological explorations would be useful to better understand the predictive factors of NC and to analyze the impact of nutrition at the onset of NC in very premature infants to optimize its prevention in the neonatal period.

## Supplementary Information

Below is the link to the electronic supplementary material.

Graphical abstract (PPTX 1.21 MB)

## Data Availability

Data is unavailable to access. The study was approved by the Ethical Committee at Hospices Civils de Lyon (HCL).

## Abbreviations

BPD: Bronchopulmonary dysplasia
BW: Birth weight
Ca: Calcium
Cr: Creatinine
GA: Gestational age
GFR: Glomerular filtration rate
IUGR: Intrauterine growth restriction
Na: Sodium
NEC: Necrotizing enterocolitis
NC: Nephrocalcinosis
P: Phosphorus
