# Supplementary material for: Nephrocalcinosis in very low birth weight infants: incidence, associated factors, and natural course
Source: Pediatr Nephrol. 2022 Mar 28;37(12):3093–104. doi: 10.1007/s00467-021-05417-w (PMC9587072; doi:10.1007/s00467-021-05417-w)
Supplement: Supplementary file 1 — Graphical abstract (PPTX 1.21 MB) [file 467_2021_5417_MOESM1_ESM.pptx]

## Slide 1
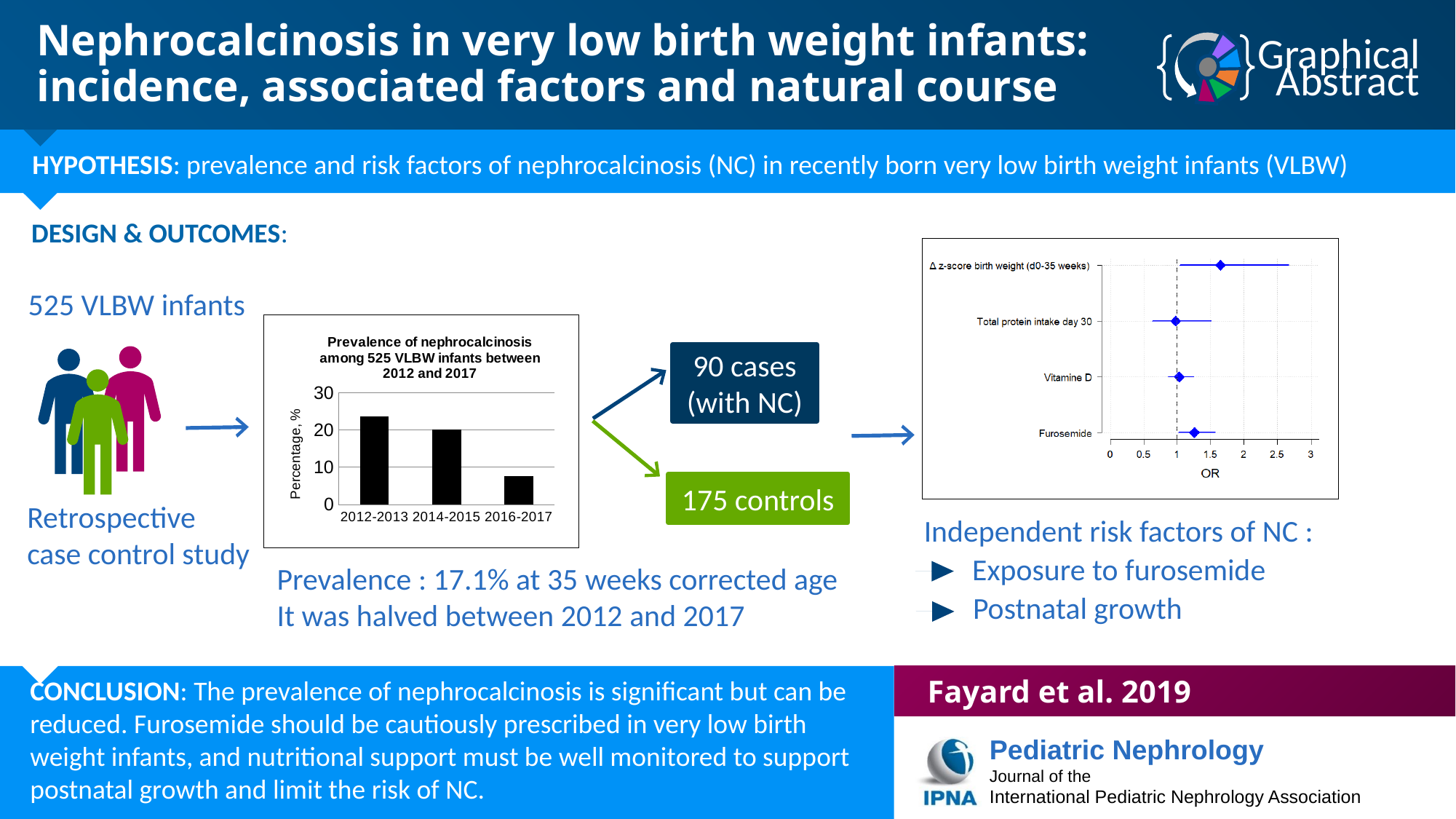

Nephrocalcinosis in very low birth weight infants:
incidence, associated factors and natural course
HYPOTHESIS: prevalence and risk factors of nephrocalcinosis (NC) in recently born very low birth weight infants (VLBW)
DESIGN & OUTCOMES:
525 VLBW infants
### Chart: Prevalence of nephrocalcinosis among 525 VLBW infants between 2012 and 2017
| Category | NC+, % |
|---|---|
| 2012-2013 | 23.6 |
| 2014-2015 | 20.2 |
| 2016-2017 | 7.6 |90 cases (with NC)
175 controls
Retrospective
case control study
Independent risk factors of NC :
Exposure to furosemide
Prevalence : 17.1% at 35 weeks corrected age
It was halved between 2012 and 2017
Postnatal growth
Fayard et al. 2019
CONCLUSION: The prevalence of nephrocalcinosis is significant but can be reduced. Furosemide should be cautiously prescribed in very low birth weight infants, and nutritional support must be well monitored to support postnatal growth and limit the risk of NC.
